# Cutaneous leishmaniasis in French Guiana: revising epidemiology with PCR-RFLP

**DOI:** 10.1186/s41182-017-0045-x

**Published:** 2017-02-28

**Authors:** Stephane Simon, Mathieu Nacher, Bernard Carme, Celia Basurko, Amaury Roger, Antoine Adenis, Marine Ginouves, Magalie Demar, Pierre Couppie

**Affiliations:** 1 Equipe EA3593 Ecosystemes Amazoniens et Pathologie Tropicale, Université de la Guyane, Campus Saint Denis, Avenue d’Estrées, 97300 Cayenne, French Guiana; 2 Centre d’Investigation Clinique Epidémiologie Clinique Antilles Guyane CIC CIE 1424, Cayenne General Hospital, 97300 Cayenne, French Guiana; 3 Laboratoire Associé – Centre National de Référence Leishmania, Laboratoire Hospito-Universitaire de Parasitologie Mycologie, Cayenne General Hospital, 97300 Cayenne, French Guiana; 4 Department of Dermatology, Cayenne General Hospital, 97300 Cayenne, French Guiana France

**Keywords:** Cutaneous leishmaniasis, *Leishmania braziliensis*, Epidemiology, PCR-RFLP, French Guiana

## Abstract

**Background:**

The development of polymerase chain reaction-restriction fragment length polymorphism (PCR-RFLP) technique for species identification among patients presenting leishmaniasis allowed to better determine the main circulating species in French Guiana.

**Methods:**

A descriptive study of the *Leishmania* species was identified, and their spatiotemporal distribution was conducted using patient records between 2006 and 2013, with 1017 new cases of leishmaniasis diagnosed. Identification was realized by PCR-RFLP on 745 cases.

**Results:**

The average proportions for different species were 86.2% for *Leishmania (Vianna) guyanensis*; 9.7% for *Leishmania (Vianna) braziliensis*; 2.8% for *Leishmania (Leishmania) amazonensis*; and 1.3% for *Leishmania (Vianna) lainsoni*, and no case of *Leishmania (Vianna) naiffi* was identified. Over this period, the proportion of cases due to *L. (V.) braziliensis* seemed to increase from 8.9% in 2006 to 13.0% in 2013 notably near the gold mining zones.

**Conclusions:**

The use of molecular tools has transformed the view of the local epidemiology of cutaneous leishmaniasis in French Guiana.

## Background

Considered like a “neglected tropical disease” by the World Health Organization [1], cutaneous leishmaniasis is a well-known parasitosis firmly established in South America [2–4]. In French Guiana, leishmaniasis was described for the first time in 1954 [5]. The annual incidence rate was estimated between 15 and 20 new cases per 10,000 inhabitants between 1979 and 2012 [6–8]. Five human *Leishmania* species have been described in French Guiana: *Leishmania (Viannia) guyanensis*, *Leishmania (Viannia) braziliensis*, *Leishmania (Leishmania) amazonensis*, *Leishmania (Viannia) lainsoni*, and *Leishmania (Viannia) naiffi* [9, 10]. These species all have a cutaneous tropism. The previous descriptions of the local epidemiology in French Guiana suggested that *L. (V.) guyanensis* is by far the predominant species [11] most frequently responsible for localized cutaneous presentations, but it may at times lead to diffuse presentations [12]. *Leishmania (Vianna) braziliensis* is a more virulent species that is potentially responsible for mucous involvement (nose, mouth, and throat). In French Guiana, there was no case caused by *L. (V.) braziliensis* described before 1986, and only nine cases have been diagnosed between 1987 and 1996 [13]. However, when these studies were undertaken, cultures were rarely performed and only when the patient’s lesion did not heal or show some clinical particularities.

The development in 2005 of a polymerase chain reaction-restriction fragment length polymorphism (PCR-RFLP) technique, by the parasitology-mycology university laboratory in Cayenne [14], allowed a more systematic species identification among patients presenting leishmaniasis lesions.

In this context, the objective of this paper is to update the epidemiological situation of leishmaniasis in French Guiana to monitor the evolution of Leishmania species and, more particularly, the emergence of *L. braziliensis* [15].

## Methods

### Objectives

This study aims to describe the spatiotemporal distribution of human cases of cutaneous leishamaniasis from the cohort of the parasitology laboratory of Cayenne General Hospital (LHUPM) in French Guiana between 1 January 2006 and 31 December 2013. The identification of *Leishmania* species corresponded to the surveillance and alert mission of the National Reference Center (CNR) of *Leishmania* for which the LHUPM constitutes a local relay.

### Type of study

The study was observational, retrospective, and monocentric.

### Study population

The study population was the patients in consultation for a suspicion of leishmaniasis at the LHUPM and in the health centers of the interior (administratively attached to Cayenne General Hospital) between 1 January 2006 and 31 December 2013. New cases were defined as cases without a history of leishmianasis in the past 12 months. Diagnosis was confirmed by the presence of *Leishmania* parasites on microscopic examination of May-Grünwald Giemsa-colored skin smears, and/or by a positive culture on RPMI culture medium, and/or by the detection of *Leishmania* DNA by PCR. During this period, 1017 cases were detected and species identification has been possible on 745 cases. This difference is explained by the fact that for some patients, we only had a stained slide, or in others, the PCR-RFLP did not allow identification (insufficient *Leishmania* DNA quantity).

### Study variables

The study variables were collected from standardized medical forms which are part of medical records both at the dermatology and the parasitology departments of Cayenne General Hospital. These variables were the following: consultation date, contamination location, profession, geographic origin, age, sex, and species of *Leishmania* identified. Presumptive contamination site was declared by the patient during the consultation. For cases diagnosed in health centers, the location was the township of the health center, unless specified otherwise.

### Leishmania identification

The species identification was performed by the University laboratory of Cayenne using a PCR-RFLP technique in collaboration with the LHUPM [14]. Briefly, DNA was extracted using DNeasy Tissue and Blood Kit (Qiagen, Hilden, Germany), according to the manufacturer’s instructions. Then, the extracted DNA was amplified with RPOF2 (5′-AGAACATGGGCGGCC-3′) and RPOR2 (5′CGAGGGTCACGTTCTTG-3′) primers (Eurogentec) which target a 615-bp region of the RNA polymerase II gene. The PCR product was digested with *TspRI* or *HgaI* (New England Biolabs). The entire reaction mixture was then analyzed by electrophoresis in a 2% agarose gel containing ethidium bromide. The resulting profile was species-specific. This identification was performed from the culture of the biopsies taken during consultations at dermatology department. If the parasite has grown, the PCR-RFLP was performed on the culture. If there was not growth, the PCR-RFLP was performed on the biopsy.

### Analysis

First, the analysis concerned the whole study population in order to conduct a quality control of the data using coherence tests and eventual queries to go back to the patient records to clean the data. After this, comparisons between proportions were performed using the chi-square test and the linear chi-square test for trend in order to identify any temporal trends. Data were analyzed with STATA 12 (College Station, Texas).

The analysis described the appropriate position and dispersion measures for quantitative variables (mean and standard deviation for Gaussian variables, median and interquartile range (IQR) for non-Gaussian variables). For qualitative variables, frequencies and proportions were reported. The calculation of the annual incidence was performed between 2006 and 2013 from the data of dermatology and the parasitology departments. Cases were reported to the total population of French Guiana using the annual data from the National Institute for Statistics and Economic Evaluation’s (INSEE) censuses and projections.

### Ethics statement

The study was retrospective. All patients were informed (leaflets, posters in several local languages) that data and analysis results may be used in research, and scientific publications, and that they had a right to refuse.

## Results

**Fig. 1:**
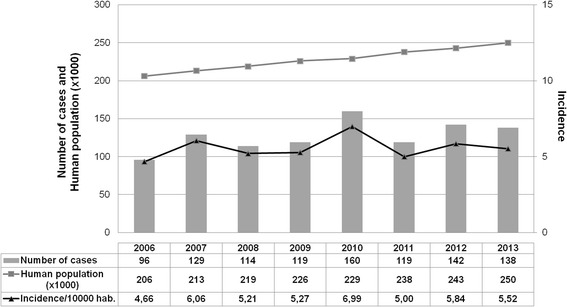
Total number of leishmaniasis cases in French Guiana (2006–2013). The total number of cases (*n* = 1017) was in relation to the number of inhabitants. The incidence rate was calculated per 10,000 inhabitants and per year

Between 1 January 2006 and 31 December 2013, 1017 new cases of leishmaniasis were diagnosed for an average of 127 cases per year, with interannual variations between 96 and 160 cases per year. The annual incidence oscillated between 4.6 and 7.0 cases per 10,000 inhabitants with an average of 5.6 cases/year (Fig. 1). The median age of patients was 35 years ranging from 3 to 87 years of age (IQR = 28–45), and the sex ratio was 4.5 (793 men/224 women). The theoretical date of infection was known in 525 cases (51.6%). The median date of infection was 1 month before the consultation, ranging from 0.25 to 360 months (IQR = 1–2). The number of lesions was known in 568 cases (55.9%). The median number was one lesion ranging from one to over 200 lesions (IQR = 1–2).

Among infected patients, 39.6% had an outdoor professional occupation (29.9% of gold miners, 22.8% of military, 14.1% of farmers, 11.4% of bricklayers, 9.2% of workers in the lumber industry); 43% had various urban or peri-urban professions; 15.3% were not employed; and 2.1% were pensioners. For 54.3% of patients, profession data was missing.

**Fig. 2:**
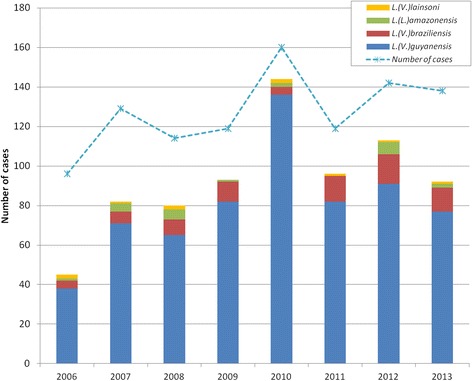
Identification of leishmania species by PCR-RFLP method on 745 samples assessed between 2006 and 2013

Concerning molecular diagnosis, 745 (73.3%) *Leishmania* species identifications were performed. The difference is explained by the fact that sometimes we receive only MGG-colored skin smears and no biopsy, but also as PCR-RFLP on biopsy is trickier (quantity of *Leishmania* DNA too low). Figure 2 illustrates the evolution of the proportion of cases by species. The average proportions were 86.2% for *L. (V.) guyanensis*; 9.7% for *L. (V.) braziliensis*; 2.8% for *L. (L.) amazonensis*; 1.3% for *L. (V.) lainsoni*; no case of *L. (V.) naiffi* leishmaniasis was identified, but the proportion of cases due to *L. (V.) braziliensis* increased from 8.9% in 2006 to 13.0% in 2013 (Table 1). Forty strains were sent to the National Reference Center for Leishmania in Montpellier (France) to confirm our diagnosis by multilocus enzyme electrophoresis (MLEE) and/or by sequencing of RNA polymerase II gene. Although *L. (V.) panamensis*, *L. (L.) colombiensis*, and *L. (L.) venezuelensis* are endemic in some countries in South America [16–18], these strains are not found in French Guiana. However, since 2007, the proportion of *L. (V.) braziliensis* has significantly increased (7.3 vs 13.3%, *P* = 0.005). The decrease observed in 2010 may have been related to small numbers which increase the risk of extreme values. The linear trend however failed to reach statistical significance (*P* = 0.07).

**Table 1:** Identification of *Leishmania* strain in French Guiana between 2006–2013.

Year	*L. (V.) guyanensis*	*L. (V.) braziliensis*	*L. (L.) amazonensis*	*L. (V.) lainsoni*
	*N* (%)	*N* (%)	*N* (%)	*N* (%)
2006 (*n* = 45)	38 (84.4)	4 (8.9)	1 (2.2)	2 (4.4)
2007 (*n* = 82)	71 (86.6)	6 (7.3)	4 (4.9)	1 (1.2)
2008 (*n* = 80)	65 (81.3)	8 (10.0)	5 (6.3)	2 (2.5)
2009 (*n* = 93)	82 (88.2)	10 (10.8)	1 (1.1)	0 (0)
2010 (*n* = 144)	136 (94.4)	4 (2.8)	2 (1.4)	2 (1.4)
2011 (*n* = 96)	82 (85.4)	13 (13.5)	0 (0)	1 (1.0)
2012 (*n* = 113)	91 (80.5)	15 (13.3)	6 (5.3)	1 (0.9)
2013 (*n* = 92)	77 (83.7)	12 (13.0)	2 (2.2)	1 (1.1)
Total (*n* = 745)	642 (86.2)	72 (9.7)	21 (2.8)	10 (1.3)

For each year, the number of cases per species is given in absolute value (*N*) and percentage (%)

**Fig. 3:**
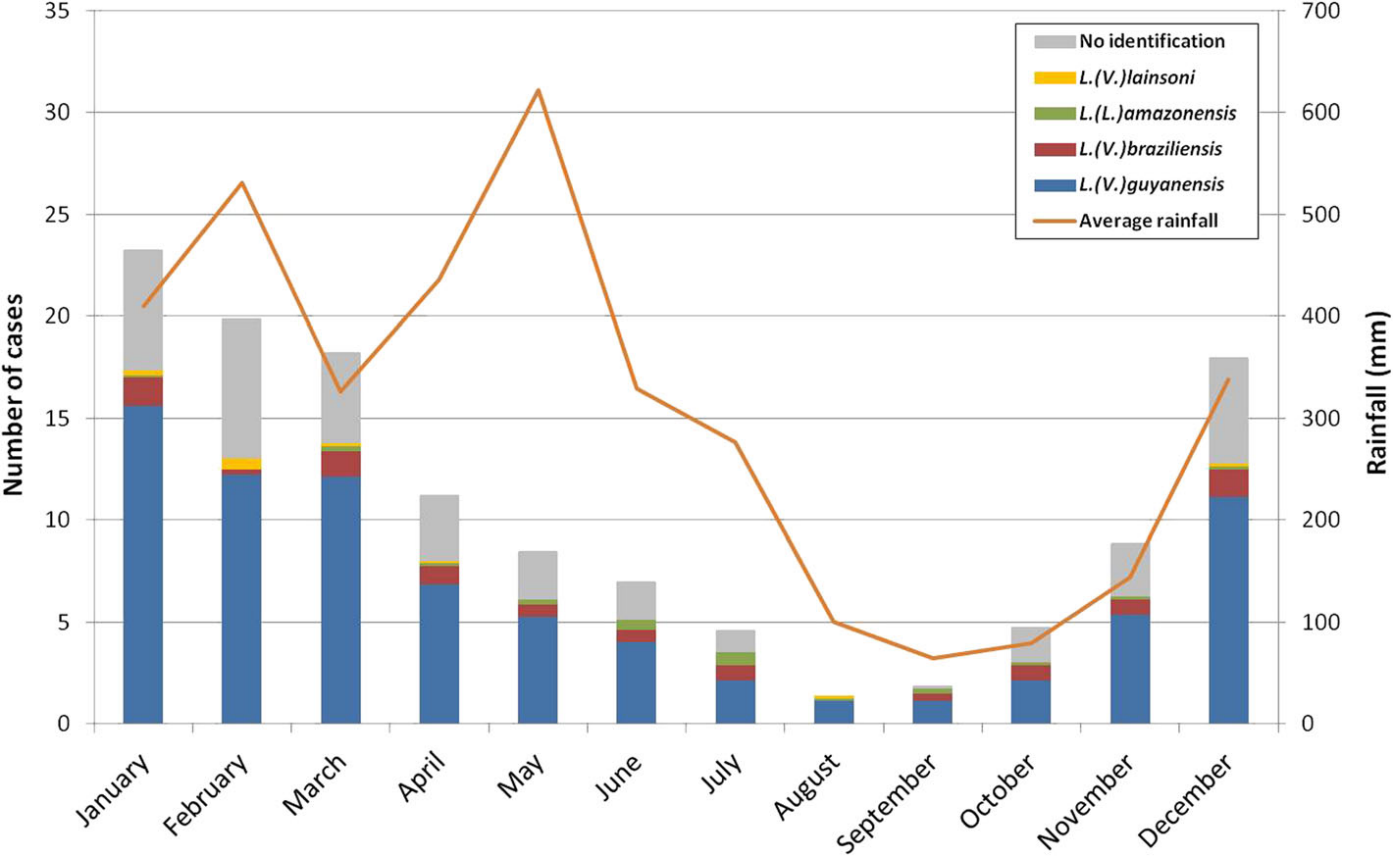
Average number of leishmaniasis cases in relation to average rainfall per month (2006–2013)

The number of monthly cases diagnosed averaged over the 8 years of the study showed a seasonal pattern with a peak in January (23.3 cases) and a minimum in August (1.4 cases) (Fig. 3).

**Fig. 4:**
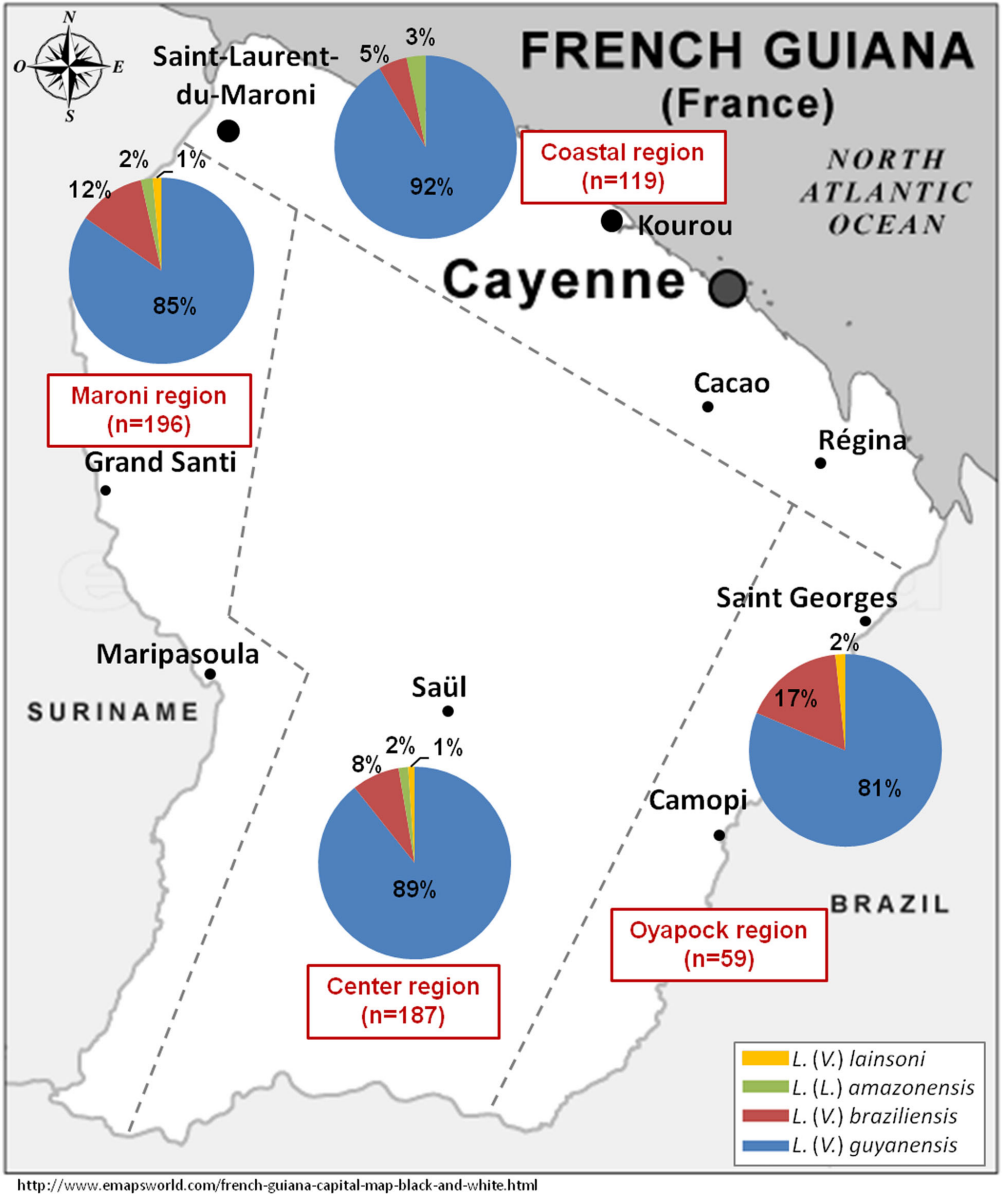
Distribution of *Leishmania* species in four major areas

The geographical distribution of 689 cases (67.7%) on the known presumptive contamination site showed that 15.4% was concentrated in the townships of Maripasoula, 9.3% in Cacao, 7.7% in Regina, 6.8% in Saül, and 5.8% in Grand Santi. On their 689 cases with a known presumptive contamination sites, species identification was possible in 561 cases. These sites were grouped into four major areas (Fig. 4). The proportion of cases due to *L. (V.) braziliensis* was higher in areas where gold mines were located.

Nine cases involved mucosal lesions: six were due to *L. (V.) guyanensis* and three cases were due to *L. (V.) braziliensis*. The frequency of mucosal forms due to *L. (V.) guyanensis* was 0.9% and 4.2% for *L. (V.) braziliensis.*

## Discussion

Data from the 1980s suggested that *L. (V.) braziliensis* did not circulate in French Guiana [19, 20]. The present results showed that this is not the case today. Moreover, during the study period, the specific incidence of *L. (V.) braziliensis* globally seemed to increase linearly. The increase of the cases due to this species could be linked to the generalization of the species diagnosis that followed from the local development of an identification technique.

Indeed, before 2006, because of the difficulties of the transatlantic transport of samples and the delays of the isoenzymes technique, the search for the causative species was mostly motivated by an unusual presentation or an unfavorable evolution. It is thus probable that cases of *L. (V.) braziliensis* leishmaniasis with a simple common cutaneous uncomplicated presentation and evolution were not identified. This hypothesis underlines the interest of the exploitation of older biological collections, which could be suitable for species diagnosis by PCR-RFLP. Another hypothesis for the apparent increase of the proportion on *L. (V.) braziliensis* would be signs of an emerging problem. This hypothesis was reinforced by the apparent preponderance of cases near gold mining sites.

The incidence calculated in the present study (5.6 cases/10,000 inhabitants/year) was four to five times lower than the values reported in the literature [6, 7]. This difference is explained by the fact that the number of cases of leishmaniasis remains stable while the population is growing rapidly (113,351 in 1990 to 250,109 in 2013). The information collected allowed us to establish a reliable indicator that shows a lower incidence during the 1990s, but a relatively stable incidence with regular seasonal variations, which still remains to be fully explained. The annual repartition of leishmaniasis diagnosis always follows a seasonal pattern as described elsewhere [21, 22], which underlines the probable influence of climate on the transmission of the parasite. The peak intensity of cases observed in 2010 may have been influenced by the El Niño phenomenon in 2009–2010 [23]. Studies have shown that this phenomenon has an impact on the increase of cases of leishmaniasis in South America [24–27]. Correlations between climatic variables, vector populations, and human contaminations have previously been described in neighboring countries [27–29]. However, local studies could be useful to complete the data with entomological studies and improve the understanding of cycles of leishmaniasis in French Guiana. It is not clear whether geographical distribution of the different *Leishmania* species is linked to different human populations or different vector populations with different competencies for different parasites.

## Conclusions

The molecular study using PCR-RFLP confirms the presence of *L. (V.) braziliensis* in French Guiana [14] with a high proportion of cases than previously thought 10% rather than absent. The use of PCR-RFLP allows rapid identification of Leishmania strains. Without treatment, the risk for patients infected with *L. (V.) braziliensis* is to develop, in some cases, the mucocutaneous form of the disease. This precose identification allows a rapid management of the patients, which limits the appearance of the cutaneomucous forms. Furthermore, species identification will be further monitored as it is not clear that the proportion of *L. (V.) braziliensis* increases significantly in the context of intense gold mining or whether we are now seeing its real impact due to the recent introduction of the PCR-RFLP technique.

## Abbreviations

IQR: Interquartile range
LHUPM: Laboratoire Hospitalo-Universitaire de Parasitologie et mycologie
